# Regulatory T cells in paracoccidioidomycosis

**DOI:** 10.1080/21505594.2018.1483674

**Published:** 2018-08-01

**Authors:** Vera L. G. Calich, Ronei L. Mamoni, Flávio V. Loures

**Affiliations:** a Department of Immunology, Institute of Biomedical Sciences, University of São Paulo (USP), São Paulo, Brazil; b Department of Morphology and Basic Pathology, Faculty of Medicine of Jundiai (FMJ), Jundiai, Brazil; c Department of Clinical Pathology, Faculty of Medical Sciences, University of Campinas (UNICAMP), São Paulo, Brazil; d Institute of Science and Technology (ICT), Federal University of São Paulo (UNIFESP) at São José dos Campos, São Paulo, Brazil

**Keywords:** *Paracoccidioides brasiliensis*, pulmonary fungal infection, Foxp3 Treg cells, T cell subsets, human paracoccidioidomycosis, murine models

## Abstract

This review addresses the role of regulatory T cells (Tregs), which are essential for maintaining peripheral tolerance and controlling pathogen immunity, in the host response against *Paracoccidioides brasiliensis*, a primary fungal pathogen. A brief introduction on the general features of Treg cells summarizes their main functions, subpopulations, mechanisms of suppression and plasticity. The main aspects of immunity in the diverse forms of the *P. brasiliensis* infection are presented, as are the few extant studies on the relevance of Treg cells in the control of severity of the human disease. Finally, the influence of Toll-like receptors, Dectin-1, NOD-like receptor P3 (NLRP3), Myeloid differentiation factor-88 (MyD88), as well as the enzyme indoleamine 2,3 dioxygenase (IDO) on the expansion and function of Treg cells in a murine model of pulmonary paracoccidioidomycosis (PCM) is also discussed. It is demonstrated that some of these components are involved in the negative control of Treg cell expansion, whereas others positively trigger the proliferation and activity of these cells. Finally, the studies here summarized highlight the dual role of Treg cells in PCM, which can be protective by controlling excessive immunity and tissue pathology but also deleterious by inhibiting the anti-fungal immunity necessary to control fungal growth and dissemination.

## Regulatory T cells – general features

Suppressor T cells were described several decades ago; however, it was only in recent years that these cells were renamed ‘regulatory T cells’ (Treg cells) and extensively studied in mice and humans. Treg cells are a fundamental arm of the immune response that control the innate and adaptive phases of immunity and play an essential role in the maintenance of self-tolerance, control of anti-tumor responses, transplantation immunity and infectious processes [1–4]. In their regulatory function, Treg cells can exert protective or deleterious effects depending on the experimental setting or disease process. By suppressing excessive immunity, Tregs can be protective by restraining tissue damage due to uncontrolled inflammation; however, the suppression of immunity can lead to uncontrolled pathogen growth or disease progression that is deleterious to the host.

**Figure 1.**
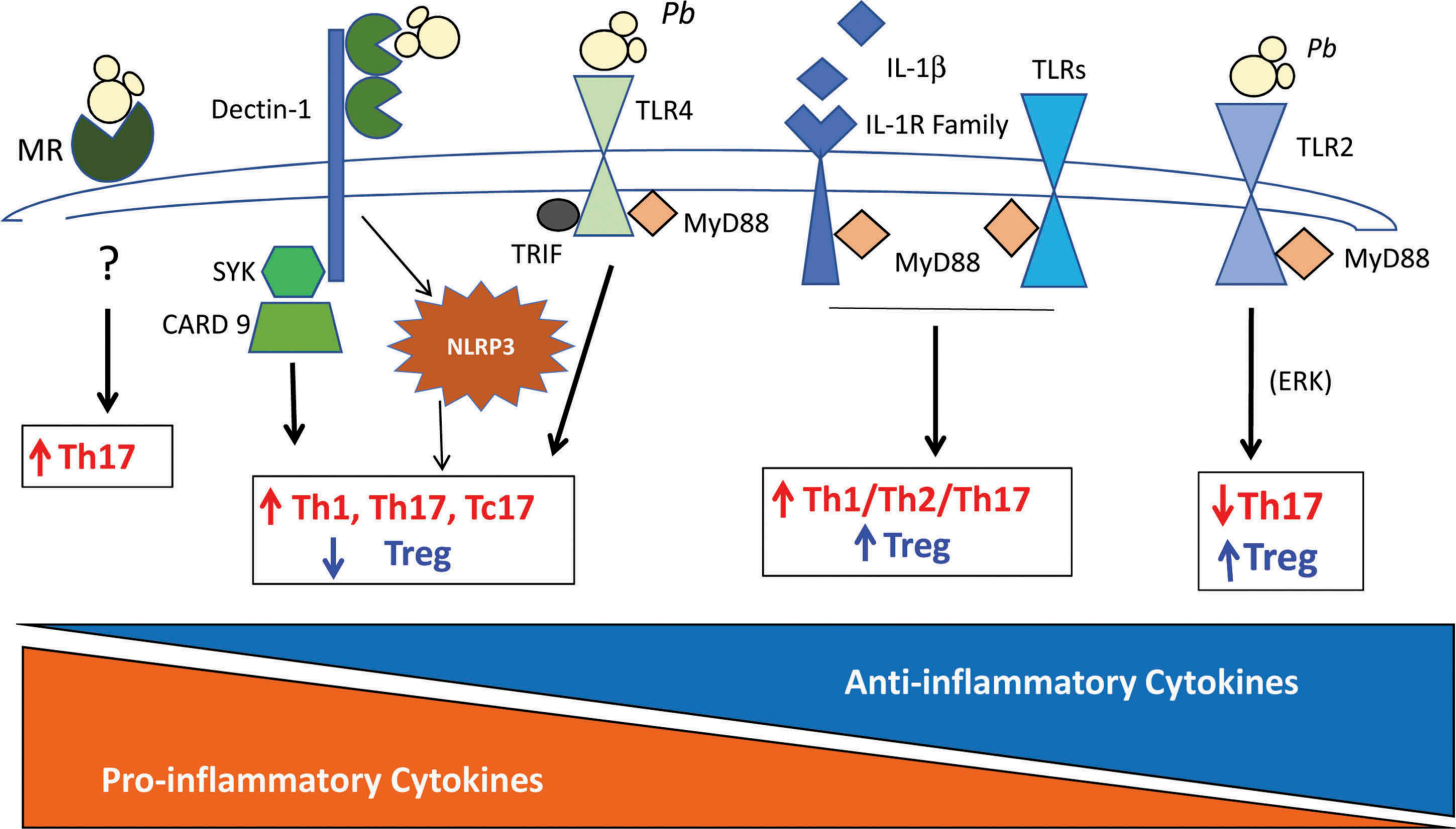
Pulmonary paracoccidioidomycosis: influence of PRRs on the expansion of Treg cells and other T cell subsets. This figure summarizes the influence of TLRs, MyD88, CLRs (dectin-1 and MR), and NLRP3 inflammasome in pulmonary PCM. Toll 2 and Toll 4 signaling exert opposing influences on T cell activation. While Toll 4 induces prevalent production of pro-inflammatory T cell subsets and reduces Treg expansion, Toll 2 signaling stimulates the prevalent differentiation of Treg cells and concomitant impairment of Th17 immunity. The adapter molecule MyD88, which controls the signaling of almost all Toll-like receptors and the IL-1 family of receptors, is fundamental to the differentiation of all T cell subsets including Treg cells. Dectin-1 via Syk – CARD9 mediated signaling is also involved in the differentiation of Th17 and Tc17 subsets with concomitant inhibition of Treg cell proliferation. MR is another CLR involved in Th17 differentiation. In pulmonary PCM, NLRP3 inflammasome mediates the prevalent differentiation of Th1 and Th17 cells and impairs Treg cell proliferation. PRRs, pattern recognition receptors; TLR2 and TLR4, Toll-like receptors 2 and 4; MyD88, myeloid differentiation factor 88; CLRs, C-type lectin receptors; MR, mannose receptor; NLRP3, NOD-like receptor P3.

There are several T cell subsets with regulatory activity; however, this review, which is not intended to be comprehensive, will focus on the two major regulatory T cell subsets, namely, natural and induced Tregs (nTreg and iTreg, respectively), that express the transcription factor Foxp3 (Forkhead box P3). The knowledge of the biology and function of Treg cells is still evolving and a recent review of Foxp3 expression and activity in Treg cells uses a diverse nomenclature for the two main subpopulations of Foxp3+ Treg cells: nTregs are designated as tTregs to define their thymic origin and pTreg to those induced at the periphery [5]. Naturally occurring Treg cells were initially described as CD4 + T cells that mature in the thymus and constitutively express CD25 (the alpha chain of IL-2R) and low levels of CD45RB. However, extraordinary advances in the knowledge of Treg cell biology came from the identification of Foxp3, a transcription factor encoded by the X chromosome that is required for the development, maintenance and function of these cells [6,7]. The importance of Foxp3 expression in the preservation of peripheral tolerance is demonstrated by the severe lymphoproliferative autoimmune diseases developed by mice (known as scurfy mice) and individuals who lack Foxp3 activity [8–10]. Scurfy mice have a spontaneous mutation of Foxp3, develop hyper-responsive CD4 + T cells and a progressive and fatal autoimmune disease [10]. Humans lacking functional Foxp3 develop early in infancy a severe and progressive autoimmune disease called IPEX, immunodysregulation, polyendocrinopathy, and enteropathy X-linked syndrome [11]. Although there is substantial evidence that Foxp3 plays a critical role in the development and function of Treg cells, there are several indications that Foxp3 expression is not sufficient for establishing the sustained suppressive function and phenotype of Treg cells [12]. Indeed, activated human conventional T cells can express low levels of Foxp3 without a suppressive function [13]. Recent reports have shown that Treg-cell-specific epigenetic changes are also critical in the process of Treg cell differentiation. The coordinated action of Foxp3 and epigenetic changes in nTregs induces a stable phenotype that sustains Foxp3 expression and their suppressive activity in several inflammatory environments and through several rounds of cell division [14]. The development of nTreg cells in the thymus requires the expression of T cell receptors (TCRs) that induce Foxp3 expression when they recognize auto-antigens in relatively high intensity, whereas TCR stimulation for an adequate length of time produces a specific Treg cell pattern of DNA hypomethylation. Both events drive thymic T cells into a stable nTreg lineage [12] the main function of which is to maintain self-tolerance. Interestingly, in vitro generated Treg cells (CD4+ CD25+ Foxp3+) present an epigenetic status that is different from nTreg, and their diverse pattern of DNA methylation confers less stable expression of Foxp3 and CTLA4 [15]. These epigenetic differences in nTreg and iTreg permit the stability of Foxp3 expression to be controlled through different groups of transcription factors [12].

In addition to CD4^+^CD25^+^Foxp3^+^ nTreg cells, induced regulatory T cells can be generated from conventional T cells under certain defined microenvironments. For example, TGF-β can convert peripheral CD4^+^CD25^−^ T cells into CD4^+^CD25^+^Foxp3^+^ suppressive iTreg cells [16]. In addition, Foxp3+ iTreg cells can differentiate in the mucosal tissue under the influence of TGF-β and retinoic acid [17]. Other suppressive T cells that do not express Foxp3 such as T regulatory-1 (TR1) and T helper-3 (Th3) can be experimentally induced, respectively, by IL-10 and TGF-ββ and their regulatory activity is mediated by the same anti-inflammatory cytokines [18].

In addition to CD25 (IL-2Rα), Treg cells express other activation markers such as CTLA-4 (CD152, cytotoxic T lymphocyte-associated antigen 4), GITR (glucocorticoid-induced tumor necrosis factor-receptor-related protein), OX40 (CD134), and L-selectin or CD62 ligand (CD62L) [19–21]. In addition to these cell markers, Treg cells were also seen to have enhanced expression of Neuropilin-1, CD39, CD73, Helios and CCR5. Although it has been postulated that Neuropilin-1 is an excellent marker for characterizing nTreg cells in diverse immune settings [22] and CD39/CD73 have been extensively used as Treg cell markers [23], as far as we know, no specific markers for nTregs or iTregs have been characterized.

Treg cells use many mechanisms to exert their suppressive effects. Their suppressive activity can be mediated by inhibitory cytokines, metabolic interference, cytolysis, and modulation of dendritic cell function. The inhibitory cytokines TGF-β, IL-10, and IL-35 are released under Treg cell stimulation and may inhibit the function of effector T cells. This inhibition can affect pro-inflammatory mechanisms mediated by Th1 and Th17 responses [24–29] as well as hyper-allergic inflammatory reactions mediated by Th2 immunity [30–32]. Surface-bound TGF-β has also been implicated in the suppressive effect of Tregs mediated by cell contact [33]. However, in some diseases and experimental settings it has been postulated that these cytokines are not needed for the suppressive function of Treg cells, indicating the relative importance of these mediators [34]. In addition to the control of adaptive immunity, these cytokines can play an inhibitory role in innate immune cells, down-modulating their inflammatory activity and their function as antigen presenting cells, an activity mainly mediated by dendritic cells (DCs).

The expression of CD25 (IL-2Rα) with high affinity for IL-2 can consume this cytokine resulting in apoptosis of actively dividing effector T cells [35]. The expression of the ectoenzymes CD39 and CD73 by Tregs catalyzes the release of extracellular adenosine, which activates the adenosine receptor (A_2A_R) and mediates immunosuppression of T cells [21]. In addition, suppressive cyclic AMP can permeate membranes of neighboring cells through gap junctions and inhibit T cell activity. Another interesting mechanism used by Treg cells is the induction of effector T cell apoptosis mediated by granzyme A or granzyme B and perforin [36]. Finally, Treg cells can modify the function of DCs by promoting their tolerogenic phenotype. The reverse signaling of CLTA4 (constitutively expressed by Tregs) on DCs via CD80/CD86 activates the expression of indoleamine 2, 3 dioxygenase (IDO), which metabolizes tryptophan into the kynurenine pathway that inhibits T cell activity and induces T cell apoptosis [37,38]. In addition, LAG3 (lymphocyte activation gene-3, CD223), a CD4 homologue expressed by Treg cells that binds MHC class II molecules with high affinity, inhibits maturation of DCs and their stimulatory activity [39]. In sum, several suppressive mechanisms can be used by Treg cells; however, the preponderant mechanism used by these cells possibly depends on the experimental setting, the tissue microenvironment and the disease process.

Finally, several studies have demonstrated that there is an integration of environmental cues by Treg cells and immune effector cells. Thus, depending on the inflammatory milieu, Treg cells can express Th lineage specific transcription factors – Tbet, IRF4, Stat3 and Bcl6 – that allow these cells to migrate and control inflammatory reactions mediated respectively by Th1, Th2, Th17 and T follicular helper cells [11].

## Treg cells can exert protective or detrimental roles in infectious diseases

The role of regulatory T cells in viral, bacterial, parasitic and fungal infections has been extensively studied, including fungal infections caused by *Candida albicans, Histoplasma capsulatum, Aspergillus fumigatus, Cryptococcus neoformans* and *Paracoccidioides brasiliensis* [40–50].

Tregs guarantee a controlled immune response upon microbial encounter, and in this manner, avoid pathological immune responses. An uncontrolled response resulting from failure to effectively control its magnitude can result in collateral injury to affected tissues and organs, also known as immunopathology. Conversely, excessive suppression generated by Tregs can compromise pathogen clearance and promote chronic infection. Thus, properly adjusted Treg function and activation is indispensable to preventing immune pathology while allowing for protective immune responses against pathogens. Several studies have shown that Tregs participate in the control of tissue damage caused by the immune system, while others have demonstrated that unbalanced effector/regulatory responses favoring Treg cells can promote pathogen persistence and chronic disease. Accordingly, high Treg cell frequency and function have been associated with impaired effector T cell activity and pathogen clearance in numerous chronic infections in mice and humans [51,52]. In some situations, Tregs are also required for long-term maintenance of protective immunity, for example, in the context of *Leishmania major* infection [53]. In rare and extreme cases, inhibition of effector responses promoted by Tregs can lead to host death, as demonstrated in the murine model of malaria caused by the parasite *Plasmodium yoelii* [54]. In contrast, in some bacterial infections such as that caused by *Mycobacterium tuberculosis*, suppression of Treg cell activity resulted in improved protective responses [55].

Many studies have concentrated on the role of Treg cells during chronic infections, but their role in acute infections was scarcely described and sometimes presented conflicting results [56]. For example, a first report demonstrated that the depletion of Tregs has an insignificant effect in the course of influenza virus infection in mice [57]. However, another study observed the emergence of antigen-specific Tregs that persisted after acute infection and were tissue protective [58].

The presence and function of Treg cells have been described in experimental models and human fungal infections. Even though a substantial part of the literature indicates that Treg cells have a detrimental effect in fungal infections, some indicate their protective activity. Indeed, in several fungal infections, including oral *C. albicans* infection, Treg cells have been reported to enhance the production of protective Th17 immunity [59]. Similarly, in murine gastric candidiasis, Treg cells reduce immunity allowing fungal survival in a controlled inflammatory environment that results in long-lasting antifungal immunity [60]. The anti-inflammatory properties of Treg cells and their ability to induce tolerance to a fungal pathogen have also been reported in candidiasis and aspergillosis [46,60]. Following the migration of Treg cells to a site of infection, Th1 cells arise and activate the indoleamine 2,3-dioxygenase (IDO) pathway of DCs via IFN-γ. The kynurenines produced enhance the differentiation of naïve T cells into Foxp3^+^ Treg cells while simultaneously restraining the differentiation of Th17 responses by inhibiting the RORγt transcription factor [61].

## Paracoccidioidomycosis: Forms of the disease and immune response

Paracoccidioidomycosis (PCM) is the most prevalent systemic mycosis in Latin America affecting immunocompetent individuals [62,63]. The incidence of the disease is very variable in different countries and even in different regions of each country. Nevertheless, the highest prevalence is reported in Brazil (80% of described cases), where some studies have estimated the incidence in endemic regions to range from 0.7 to 3.7 cases/100,000 inhabitants/year [62,64–67]. Caused by dimorphic fungi of the *Paracoccidioides* genus (*P. brasiliensis* and the recently identified *P. lutzii*) [64,67], the infection occurs by the respiratory route after conidia inhalation [68]. In lungs, the fungal propagules can be contained and destroyed by resident innate immune cells, can cause localized lesions, or can disseminate via the hematogenous or lymphatic system [68].

An infection with *P. brasiliensis* can present three outcomes: 1) an asymptomatic infection (named PCM-infection (PI)), common in individuals who live or work in endemic areas, identified by positive delayed-type hypersensitivity (DTH) skin tests to fungal antigens, but no symptoms of the disease are presented; 2) the acute/subacute form (AF – formerly called juvenile form), which generally affects children and young adults of both sexes and is characterized by rapid fungal dissemination and involvement of the lymph nodes, liver, spleen and bone marrow; and 3) the chronic form (CF – formerly adult form), mainly observed in older individuals, predominantly men, presenting heterogeneous clinical manifestations, ranging from isolated pulmonary or epithelial lesions (unifocal form) to systemic involvement (multifocal form) [62,65,66,69–74].

The acquired immune response pattern elicited after infection is believed to influence the disease’s evolution and clinical manifestations. AF is distinguished by predominant Th2/Th9 cell activation [75] and increased production of cytokines such as IL-4, IL-5, IL-9, IL-10, TGF-β, and IL-27, as well as low production of IFN-γ and TNF-α [75–77]. Concomitantly, AF patients present polyclonal activation of B cells [78] and produce high amounts of specific IgG4 and IgE antibodies [79–81]. CF patients develop a mixed immune response with the predominant differentiation of Th17/Th22 cells, high production of IL-17 and IL-22 [75], and elevated levels of specific IgG1 antibodies [79–81]. Furthermore, cells from these patients are also able to produce Th1-type cytokines such as IFN-γ, TNF-α, and IL-2 and variable amounts of IL-10 and IL-4 [75–77]. In contrast, cells from individuals presenting the asymptomatic infection (PI) respond *in vitro* to *P. brasiliensis* stimulus, differentiating into Th1 cells and producing high amounts of Th1 cytokines (IFN-γ and TNF-α) [75,77]. The immune responses found in the main clinical forms of PCM resemble those described in the experimental murine model of susceptibility and resistance to the disease [82–85].

## 4- Regulatory T cells in the human PCM

Impairment of the cellular immune response in PCM patients, as evidenced by DTH anergy and decreased lymphocyte proliferation in response to fungal antigens, has been noted in studies since the 1970s [86–88]. The mechanisms involved in this immunosuppression are not yet fully understood but appear to be related to an imbalance in the production of suppressive cytokines such as IL-10 and TGF-β, high expression of CTLA-4 (CD152) in circulating lymphocytes [89], and the increased apoptosis rate of effector T cells via the CD95-CD95L (Fas-FasL) interaction [89]. Altogether, these characteristics suggest the participation of regulatory T cells (Tregs) in the development and control of human PCM.

Indeed, the presence of cells expressing Foxp3 in the inflammatory infiltrate of lesions caused by *P. brasiliensis* has been shown, particularly in CF patients [75,90]. Additionally, a positive correlation has been found between the number of Foxp3^+^ cells and the density of *P. brasiliensis* yeast cells in oral mucosa lesions [91]. Furthermore, TCD4^+^CD25^+^ cells isolated from PCM patients’ lesions present strong *in vitro* regulatory activity, diminishing the proliferative response of TCD4^+^CD25^−^ cells [45].

In addition to the presence of Treg cells in *P. brasiliensis-*induced lesions, PCM patients present an increased frequency of CD4^+^CD25^+^Foxp3^+^ cells in peripheral circulation, which returns to levels comparable to healthy subjects after effective anti-fungal treatment [90]. Furthermore, circulating CD4^+^CD25^+^Foxp3^+^ cells of PCM patients can exhibit high surface expression of molecules associated with Treg functions such as CTLA-4, LAP-1 (latency-associated peptide (TGF-β)), and GITR [45,90].

As previously described, Treg cells can exert their functions through several mechanisms [92], and *in vitro* studies with Treg cells isolated from peripheral blood of PCM patients showed that both contact-dependent suppression and the production of soluble factors can be part of their actions [45,90]. The contact-dependent suppression of the immune response observed in PCM may be partially due to CTLA-4 signaling since mononuclear cells from PCM patients recover their proliferative capacity and increase their production of IFN-γ when CTLA-4 is blocked by neutralizing antibodies *in vitro* [89]. These data are in agreement with other findings demonstrating that CTLA-4 expressed by Treg cells interacts with its ligands (CD80 and CD86) in antigen-presenting cells (DCs and macrophages), thereby inducing the production of IDO. This enzyme is a potent inhibitory molecule that acts on the induction of pro-apoptotic metabolites via tryptophan metabolism, resulting in the suppression of effector T cells [37,93]. In addition, the interaction of CTLA-4 expressed by Treg cells with CD80 and CD86 molecules on antigen-presenting cells competes with the binding of the CD28 molecules expressed on effector T cells, preventing their complete activation [92,94]. Moreover, signaling via CTLA-4 increases the availability of membrane-associated TGF-β in the region of contact between Treg and effector cells, enhancing its suppressive activity [95]. Treg cells can also act by inducing the apoptosis of effector T cells directly via CD95-CD95L interaction [96,97]. In human PCM, Campanelli *et al*. (2003) showed that blockade of this interaction resulted in a significant reduction of the apoptosis rate of T cells *in vitro* [89].

Lastly, the production of suppressive cytokines such as TGF-β (soluble or membrane-associated with LAP-1) and IL-10 by Treg cells can contribute to the suppression of the cellular immune response [98]. As mentioned, CD4^+^CD25^+^Foxp3^+^ cells of PCM patients present increased expression of LAP-1 [45,90], and CD4^+^CD25^+^ cells purified from peripheral blood of PCM patients with active disease produce high amounts of IL-10 and soluble TGF-β when stimulated *in vitro* [90]. Blockade of these cytokines reverted the proliferative response of CD4^+^CD25^−^ cells from PCM patients co-cultured with CD4^+^CD25^+^ cells [90].

TGF-β is a pleiotropic cytokine with functions including the induction of tolerance-regulating lymphocyte activation, differentiation, proliferation, and survival [99,100]. As commented before, besides its role in the suppression of effector T cell activation, TGF-β is necessary for the differentiation and expansion of iTregs [101]. It can also modulate, in conjunction with IL-10, the inflammatory response by contributing to the inactivation of inflammatory macrophages and initiating tissue healing and fibrosis by inducing the production of extracellular matrix components by fibroblasts [99,100]. Consequently, in addition to inducing the direct suppression of effector T cells, IL-10 and TGF-β production by Treg cells also contribute to the modulation of the immune response by inducing the differentiation and expansion of Treg cells.

Although our knowledge about the mechanisms through which Treg cells exert their regulatory effects in human PCM is growing but still limited, the experimental model of *P. brasiliensis* infection confirmed some of the above-mentioned findings and expanded our comprehension about the role of Treg cells in the development of the disease.

## Regulatory T cells in murine PCM

An initial study by our group demonstrated that Treg cells exert a deleterious effect on mice resistant (A/J) or susceptible (B10.A) to *P. brasiliensis* infection. Depletion of Treg cells by an anti-CD25 monoclonal antibody led to less severe and regressive infection in addition to decreased tissue pathology in both mouse strains. Anti-CD25 treatment reversed the initial T cell anergy of the resistant A/J mice and enhanced their late and protective Th1 and Th17 responses against the fungus. In the susceptible B10.A mice, the reduction in Treg cells led to efficient immunity, rescued the late production of Th1, Th2, and Th17 cytokines, and more importantly, abolished the increased mortality of this mouse strain [43].

Further studies in the murine model provided evidence for the dual role of Treg cells in the severity of pulmonary PCM [44]. Using a loss- and gain-of-function experimental approach for the manipulation of Treg cells *in vivo*, a role of Treg cells in the inhibition of both the protective and deleterious aspects of the immune response against *P. brasiliensis* infection was demonstrated. Using Foxp3^GFP^ transgenic mice it was shown that natural Tregs migrated to the lungs of *P. brasiliensis* infected mice at the early and late periods of infection, where these cells became activated. The depletion of Treg cells with anti-CD25 antibodies increased Th1, Th2, and Th17 immunity as previously demonstrated by Felonato et al. [43]. This increased T cell immunity promoted a reduced fungal load in the lungs and diminished pathogen dissemination to other organs such as the liver and spleen. Importantly, after adoptive transfer of T cells using immunodeficient Rag1^−/-^ mice, it was found that isolated CD4^+^Foxp3^+^ Treg cells were able to confer some degree of protection to infected mice and that conventional CD4^+^Foxp3^−^ T cells alone, despite their contribution to fungal elimination and enhanced T cell immunity, induced increased inflammatory reactions in the lungs. Importantly, the transfer of Tregs combined with CD4^+^Foxp3^−^T cells generated a more efficient and well-adjusted immune response able to limit pathogen growth and excessive tissue inflammation, leading to less severe disease and higher survival rates [44]. This study also shed light on the function of Tregs in human PCM, since to our knowledge, all published works have associated the presence of these cells with the severe forms of the disease. However, the improvement of some PCM patients is only obtained when the anti-fungal therapy is associated with anti-inflammatory drugs, pointing out the importance of controlled inflammation as a therapeutic tool [68,102]. Thus, further studies on the role of Tregs in PCM are necessary to better understand the function of the several T cell subsets in the human disease.

The harmful role of Treg cells in pulmonary PCM has been previously demonstrated using CD28^−/-^ mice. Despite their early protective immunity, CD28-sufficient mice showed at the late phase of infection a persistent presence of Treg cells, impaired T cell responses, elevated production of anti-inflammatory cytokines and higher mortality rates when compared with CD28^−/-^ mice [103]. These data are supported by another study using CCR5^−/-^ mice. The migration of Treg cells is dependent on the CCR5-CCR5R axis, and CCR5 ligands were shown to be produced in the lesions of *P. brasiliensis* infected mice. Therefore, CCR5^−/-^ mice were found to exhibit decreased frequency of Tregs in granulomatous lesions and consequently reduced fungal growth and dissemination in relation to their WT counterparts. In the absence of CCR5, the granulomas presented a reduced frequency of CD4^+^CD25^+^Foxp3^+^ Tregs and GITR and CTLA-4 expressing T cells [104].

In agreement with the findings in the human disease, the great majority of experimental models of murine PCM has described the association of Treg cells with more severe disease. The depletion of PMN leukocytes in ongoing pulmonary PCM of BALB/c mice led to less severe infection and reduced inflammation containing diminished numbers of Treg cells but augmented levels of some anti-fibrotic mediators able to control lung fibrosis, inhibiting a typical response of that mouse strain [105]. In accordance, the expression of the enzyme 5-lipoxygenase was shown to be detrimental to systemic PCM because it reduced Th1 immunity and increased the presence of Treg cells that allowed fungal growth and appeared to have contributed to the of non-organized inflammatory reactions [106]. Another study investigating the role of B-1 cells in pulmonary PCM showed their deleterious effect, which was attributed to the increased migration and activation of Treg cells [107]. In contrast with those previously described deleterious effects of Treg cells, a study employing a repeated immunization with a DNA vaccine encoding the immunoprotective peptide (P10) from gp43, the main diagnostic antigen of *P. brasiliensis*, conferred a long-term protection to infected mice by inducing increased numbers of memory and regulatory T cells able to control the infectious process and the inflammatory tissue pathology [108].

Several reports brought evidence that the expansion of Treg cells in the pulmonary PCM is associated with the expression and activity of some components of the innate immune system. In the pulmonary model of PCM, it was demonstrated that Toll-like receptors (TLRs), C-type lectin receptors (CLRs), NOD-like receptors and the MyD88 adapter molecule participate in the control of Treg cell expansion [109–113]. In a first study using TLR2^−/-^mice, a diminished number of Treg cells was associated with uncontrolled inflammatory responses and increased tissue pathology, suggesting a protective role of these cells [109]. Using another experimental model, it was seen that in comparison with TLR4^−/-^, TLR4-sufficient mice presented reduced proliferation of Treg cells, an augmented influx of activated T lymphocytes and macrophages to the lungs and a more severe fungal infection than TLR4-deficient mice. The divergent patterns of immunity developed by TLR4^−/-^ and TLR4-sufficient mice, however, resulted in equivalent mortality rates, indicating that the control of elevated fungal growth mediated by vigorous inflammatory reactions is as deleterious to the host as a low fungal load inefficiently controlled by limited inflammatory reactions [110].

Sutmuller et al. [42] were the first to demonstrate that Treg cell expansion and function are dependent on TLR2 and MyD88 signaling. In murine PCM, the absence of MyD88 signaling resulted in highly increased mortality rates in *P. brasiliensis* infected mice. The severe disease developed by MyD88^−/-^ mice was associated with uncontrolled fungal growth, impaired T cell immunity, and the absence of organized granulomatous lesions. MyD88-mediated signaling was shown to be necessary to mount protective Th responses, including Th17 immunity that promotes PMN-rich inflammatory reactions, which are able to restrain fungal growth and dissemination into other organs and tissues. Using a MyD88-dependent pathway, WT mice expanded the Foxp3^+^ Treg subpopulation that is able to control adaptive immunity while avoiding excessive inflammation. This well-balanced immunity mediated by Th1, Th2, and Th17 cells appeared to control fungal growth without significant tissue damage, which led to an extended survival time in WT mice. In contrast, the absence of MyD88 signaling appears to profoundly suppress the development of adaptive immunity, as shown by decreased levels of Th1, Th2, and Th17 cytokines, suppressed lymphoproliferative activity, and diminished activation and migration of mononuclear phagocytes and T cells (CD4^+^ and CD8^+^) to the site of infection. This defective innate immunity and impaired adaptive immunity, including deficient Treg expansion, resulted in uncontrolled fungal growth, which contributed suggestively to the tissue pathology observed in MyD88^−/−^ mice [111].

In murine PCM, the CLR dectin-1 seems to be involved in the induction and modulation of adaptive immunity, and its expression contributes to the development of efficient T-cell immunity controlled by moderate expansion of Treg cells. Indeed, *P. brasiliensis* infected dectin-1^−/−^mice were found to develop less organized and more severe lesions than their normal counterparts. This pattern of inflammation was associated with elevated fungal burden at the site of infection that caused increased fungal dissemination to several organs and tissues, possibly contributing to the increased mortality rates of dectin-1^−/−^ mice. Moreover, the reduced activation of effector/memory CD4^+^ and CD8^+^ T cells associated with increased numbers and frequency of CD4^+^CD25^+^Foxp3^+^ Tregs at the site of infection demonstrated the important role of dectin-1 in defining the severity of pulmonary PCM [112].

The NOD-like receptor P3 (NLRP3) inflammasome is an intracellular multimeric complex that triggers the activation of inflammatory caspases and the maturation of IL-1β and IL-18, important cytokines for the innate immune response against pathogens. A pioneering study by Tavares et al [113] has shown that *P. brasiliensis* activates IL-1β production dependent on the activation of NLRP3, a cytosolic PRR. Another work using the systemic route of infection [114] has demonstrated that IL-18, secreted under the influence of NLRP3 activation, was involved in Th1 cell-mediated immunoprotection of *P. brasiliensis* infected mice. Unfortunately, the behavior of Treg cells was not evaluated in this study. Our *in vivo* studies using C57BL/6 mice deficient for several NLRP3 inflammasome components (Nlrp3^−/−^, Casp1/11^−/−^, Asc^−/−)^ as well as for ATP receptor (P2x7r^−/−^) i.t infected with *P. brasiliensis* yeasts revealed that the activation of the NLRP3 inflammasome has an important role in immunoprotection against pulmonary PCM by promoting the expansion of Th1/Th17 immunity and reducing the suppressive control mediated by Treg cells [115].

Figure 1 depicts the main PRRs involved in *P. brasiliensis* recognition and their influence on the expansion of Treg cells and other T cell subsets. The individual contribution of each of those receptors can be seen, which, in some instances, exert opposing influences on the activation of innate and adaptive immunity. These findings raised an important question: which of those receptors are more important for determining the pattern of immune response that follows *P. brasiliensis* infection? The answer is complex, but our studies with two mouse strains with polar genetic susceptibility to this pathogen have demonstrated that each genetic pattern expresses different levels of these receptors, and it is the concerted action of them and their signaling pathways that determines the final configuration of the innate response and the subsequent adaptive immunity and infection outcome.

Indoleamine 2,3-dioxygenase (IDO), an enzyme mainly induced by IFN-γ, catabolizes tryptophan along the kynurenines pathway as described above. In infectious processes, IDO activity causes reduced pathogen growth due to nutrient starvation, and reduced inflammatory reactions linked to the activity of kynurenines that suppress immune responses. IDO-expressing antigen-expressing cells (APCs) play potent regulatory roles by blocking T cell responses and inducing naïve T cells to differentiate into Tregs [38,116]. Previous reports showed that IDO plays an important role in the immunity against commensal fungi such as *A. fumigatus* and *C. albicans* by regulating fungal tolerance mediated by the enzymatic activity of IDO, tolerogenic DCs and expansion of Treg cells [117–119]. The role of IDO in pulmonary PCM was first investigated using resistant (A/J) and susceptible (B10.A) mice. In both mouse strains, IDO blockade by 1-methyl tryptophan resulted in inefficient fungal clearance accompanied by enhanced T cell immunity. Despite these equivalent biological effects, only in susceptible mice did IDO inhibition cause progressive fungal growth and tissue pathology resulting in increased mortality. Despite the diverse disease outcomes, IDO inhibition induced some phenotypes that were shared by resistant and susceptible mice: the increased migration of DCs and CD8^+^ and CD4^+^ T cells to the site of infection concomitantly with reduced influx of Treg cells. In addition, IDO inhibition led to increased TGF-β and IL-6 synthesis, enhancing Th17 differentiation and reducing the expansion of Th1 and Treg cells [120]. These findings are in agreement with an elegant study showing that IDO activates regulatory T cells and blocks their conversion into Th17 cells [116].

A key role of IDO and Treg cells was also demonstrated during an investigation of the contribution of plasmacytoid dendritic cells (pDCs) to host defenses against murine PCM. In this model, tolerogenic activity of pDCs was described associated with increased expansion of Treg cells possibly by an IDO-mediated mechanism. The depletion of pDCs using monoclonal antibodies resulted in a less severe disease and a high frequency of activated CD4^+^ and CD8^+^ T lymphocytes, concomitant with elevated numbers of macrophages and neutrophils that migrate to the lungs of depleted mice. Furthermore, a reduced number of Foxp3^+^ Treg cells and decreased presence of pDCs expressing IDO were found in the lungs of pDC-depleted mice. Strong evidence for the role of IDO expression in the expansion of Treg cells was obtained using an *in vitro* experiment: pDCs of IDO-deficient mice and 1MT-treated pDCs of WT mice stimulated by *P. brasiliensis* yeast were more efficient at the induction and activation of T cells with concomitant reduction of Treg cells [121]. No surprises came out when we further explored the regulatory mechanism exerted by IDO-1 in the early and late phases of immunity against *P. brasiliensis* infection using IDO-1^−/-^ mice. The absence of IDO-1 expression increases fungal burden, potentiating the expression of activation markers in macrophages and DCs resulting in exacerbated Th17 immunity poorly controlled by Treg cells. This type of immunity is the worst scenario for a host response to a pathogen, where uncontrolled fungal growth is accompanied by intense but inefficient inflammatory reactions causing exaggerated tissue pathology that ultimately leads to the accelerated death of infected mice [122]. Both studies showed that the IDO/Treg/Th17 axis plays important roles in the control of immunity and the severity of pulmonary PCM [121,122].

Another recent study by our group has demonstrated that in pulmonary paracoccidioidomycosis IDO exerts a dual function depending on the resistance pattern of the host. IDO activity is predominantly enzymatic and induced by IFN-γ signaling in pulmonary dendritic cells (DCs) from infected susceptible (B10.A) mice, whereas phosphorylated IDO (pIDO) triggered by TGF-β activation of DCs functions as a signaling molecule in resistant mice. IFN-γ signaling activates the canonical pathway of NF-κB, which promotes a pro-inflammatory phenotype in B10.A DCs that controls fungal growth but ultimately suppresses T cell responses. In contrast, in A/J DCs phosphorylated IDO (pIDO) promotes a tolerogenic phenotype that conditions sustained synthesis of TGF-β and expansion of regulatory T cells and avoids excessive inflammation and tissue damage contributing to host fitness. Taken together, our studies on the influence of IDO on PCM clearly demonstrated its crucial importance for the induction of regulatory T cells. Moreover, we have been able to clarify the two IDO-dependent mechanisms that trigger the proliferation of Treg cells: one through its enzymatic activity and production of kynurenines induced by IFN-γ and the other through its signaling function mediated by TGF-β-induced pIDO. This latter mechanism is a potent inducer of tolerogenic DCs that can contribute to fungal resistance by means of disease tolerance, a mechanism that preserves host fitness instead of pathogen clearance [123].

## Concluding remarks

Many recent studies have contributed to a better understanding of the biology of Treg cells and their control of the immune system by inducing auto-tolerance and regulating immunity against endogenous and exogenous insults. This information has also improved our understanding of immunological mechanisms that confer protection against infectious agents, including *P. brasiliensis*. However, studies on the role of Treg cells in human PCM have been scarce, and most of them point to the deleterious effect Treg cells have through their inhibition of protective adaptive immunity. Nevertheless, several studies using murine models of infection have demonstrated that various mechanisms mediated by components of innate immunity have crucial importance in the development of Treg cells and their control of adaptive immunity. The dual role of Treg cells has been clearly demonstrated; they may exert protective mechanisms by controlling excessive inflammation and tissue pathology, but they may also act deleteriously by preventing the development of efficient immune responses that control fungal growth and dissemination. Further studies on the role of Treg cells in experimental models and in the human disease will certainly contribute to a better characterization of the immunopathology of PCM leading to more safe and efficient therapeutic procedures.
